# A linguistic rule-based approach to extract drug-drug interactions from pharmacological documents

**DOI:** 10.1186/1471-2105-12-S2-S1

**Published:** 2011-03-29

**Authors:** Isabel Segura-Bedmar, Paloma Martínez, César de Pablo-Sánchez

**Affiliations:** 1 Computer Science Department, University Carlos III of Madrid, Leganés, 28911, Spain

## Abstract

**Background:**

A drug-drug interaction (DDI) occurs when one drug influences the level or activity of another drug. The increasing volume of the scientific literature overwhelms health care professionals trying to be kept up-to-date with all published studies on DDI.

**Methods:**

This paper describes a hybrid linguistic approach to DDI extraction that combines shallow parsing and syntactic simplification with pattern matching. Appositions and coordinate structures are interpreted based on shallow syntactic parsing provided by the UMLS MetaMap tool (MMTx). Subsequently, complex and compound sentences are broken down into clauses from which simple sentences are generated by a set of simplification rules. A pharmacist defined a set of domain-specific lexical patterns to capture the most common expressions of DDI in texts. These lexical patterns are matched with the generated sentences in order to extract DDIs.

**Results:**

We have performed different experiments to analyze the performance of the different processes. The lexical patterns achieve a reasonable precision (67.30%), but very low recall (14.07%). The inclusion of appositions and coordinate structures helps to improve the recall (25.70%), however, precision is lower (48.69%). The detection of clauses does not improve the performance.

**Conclusions:**

Information Extraction (IE) techniques can provide an interesting way of reducing the time spent by health care professionals on reviewing the literature. Nevertheless, no approach has been carried out to extract DDI from texts. To the best of our knowledge, this work proposes the first integral solution for the automatic extraction of DDI from biomedical texts.

## Background

A DDI occurs when one drug influences the level or activity of another, for example, raising its blood levels and possibly intensifying its side effects or decreasing drug concentrations and thereby reducing its effectiveness. The detection of DDI is an important research area in patient safety since these interactions can become very dangerous and increase health care costs. Although there are different databases supporting health care professionals in the detection of DDI, these databases are rarely complete, since their update periods can reach three years [1]. Drug interactions are frequently reported in journals of clinical pharmacology and technical reports, making medical literature the most effective source for the detection of DDI. Thus, the management of DDI is a critical issue due to the overwhelming amount of information available on them [2].

Information Extraction (IE) can be of great benefit in the pharmaceutical industry allowing identification and extraction of relevant information on DDI and providing an interesting way of reducing the time spent by health care professionals on reviewing the literature. Moreover, the development of tools for automatically extracting DDI is essential for improving and updating the drug knowledge databases. Nevertheless, no approach has been carried out to extract DDI from biomedical texts.

Most research has centered around biological relationships (genetic and protein interactions (PPI)) due mainly to the availability of annotated corpora in the biological domain, a fact that facilitates the evaluation of approaches. In general, current approaches can be divided into three main categories: linguistic-based, pattern-based and machine learning-based approaches.

The general idea of *linguistic-based approaches* is to employ linguistic technology to grasp syntactic structures or semantic meanings that could be helpful to discover relations from unstructured texts. *Pattern-based approaches* design a set of domain-specific rules (also called patterns) that encode and capture the various forms of expressing a given relationship. As opposed to the previous approaches, which need a laborious effort to define grammars or a set of rules, the *machine learning* methods allow to automatically acquire and code all the necessary knowledge. Table 1 shows some of the main works for biomedical relation extraction.

**Table 1:** Main approaches for PPI extraction

System	Approach	Corpus	F_1_
*IntEx*[ 28]	Link grammar + patterns	DIP	38.9%
*AkanePPI *[29]	dependency parsing + pattern matching	BioCreative-PPI	19%
Verspoora et al. [30]	semantic grammar + pattern matching	BioCreative-PPI	25.2%
BioPPISVMExtractor [31]	Link grammar parser + SVM^1^	DIP	57.85%
Chen et al. [32]	SVM	BioCreative-PPI	57.8%
Airola et al., [33]	dependency-path kernel	Aimed, BioInfer, HPRD50, IEAP, LLL	56.4% (AIMed)

The comparison among different works is not always possible because many of them have been evaluated on different corpora. Therefore, it is risky to draw conclusions on the performance of the different techniques. In general terms, the linguistic-based approaches perform well for capturing relatively simple binary relationships between entities in a sentence, but fail to extract more complex relationships expressed in various coordinate and relational clauses [3]. We believe that the performance of linguistic-based approaches is strongly influenced by the shortage of biomedical parsers. General purpose parsers, which have been trained on generic newswire texts, are not able to deal with the complexity of the biomedical sentences that tend to cause problems due to their length and high degree of ambiguity [4].

Pattern-based approaches usually achieve high precision, but low recall. They are not capable of handling long and complex sentences, so common in biomedical texts. Furthermore, these approaches are limited by the extent of the patterns, since relations spanning several sentences cannot be detected by them. Linguistic phenomena including modality and mood, which can alter or even reverse the meaning of the sentence, have hardly ever been studied by the pattern-based approaches. Thus, pattern-based approaches are not able to correctly process anything other than short and straightforward sentences [3], which, on the other hand, are quite rare in biomedical texts.

In general, machine learning-based approaches have achieved better performance than linguistic-based and pattern-based ones, as demonstrated in the last BioCreative challenge [5]. One important advantage of these approaches is that they can be easily extended to new set of data or a new task or domain. However, machine learning-based approaches depend heavily on the annotated corpora for training and testing. Corpus annotation is an expensive work, usually involving an extensive time and labor.

Although many approaches have been proposed to extract biomedical relations, only a few of them achieve successful results. One important reason is that only a few approaches have dealt with the issue of the complexity of biomedical sentences [6]. However, language structures such as apposition, coordination and complex sentences are very common in the biomedical literature. We think that the detection of these linguistic phenomena is essential to successfully tackle the extraction of biomedical relations, in particular, DDI.

**Figure 1:**
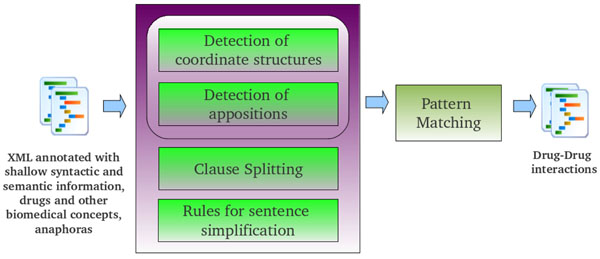
**Architecture for drug-drug interactions extraction.** This figure shows the pipeline architecture of our drug-drug interaction prototype. Firstly, texts are processed by the MMTx program. This tool performs sentence splitting, tokenization, POS-tagging, chunking, and linking of phrases with UMLS concepts. Then, appositions and coordinate structures are interpreted based on shallow syntactic parsing provided by the UMLS MetaMap tool (MMTx). Subsequently, complex and compound sentences are broken down into clauses from which simple sentences are generated by a set of simplification rules. Finally, the lexical patterns are matched with the generated sentences in order to extract DDIs.

In this work, we propose a hybrid method that combines shallow parsing and pattern matching to extract relations between drugs from biomedical texts (see Figure 1). A pharmacist defined a set of domain-specific lexical patterns to capture the most common expressions of DDI in texts, based on her professional experience and the corpus observation. Our method is based on the approach described in [6], which proposes a set of syntactic patterns to split the long sentences into clauses from which relations are extracted by a pattern matching algorithm. This approach works on the detection of appositions, coordinate constructions and relative clauses. Our contribution extends this approach dealing with any kind of subordinate and coordinate clause. Appositions and coordinate structures are interpreted based on shallow syntactic parsing provided by the UMLS MetaMap tool (MMTx) [7]. Subsequently, complex and compound sentences are broken down into clauses from which simple sentences are generated by a set of simplification rules. Finally, the lexical patterns are matched with the generated sentences in order to extract DDI.

## Methods

### The DrugDDI Corpus

Most biomedical corpora (BioInfer [8], BioCreAtIvE-PPI [9] or AIMed [10]) have focus on describing genetic or protein interactions, but none contains DDI. While NLP techniques are relatively domain-portable, corpora are not [11]. For this reason, we have created the first annotated corpus that studies the phenomena of interations among drugs.

The DrugDDI corpus consists of 579 documents describing DDI. These documents were randomly selected from the DrugBank database [12] and analyzed by the UMLS MetaMap Transfer (MMTx) tool [7] that performs sentence splitting, tokenization, POS-tagging, shallow syntactic parsing, and linking of phrases with Unified Medical Language System (UMLS) Metathesaurus concepts. Thus, MMTx allows to recognize a variety of biomedical entities, including drugs. The DrugDDI corpus consists of 66,021 phrases from which 22.6% (14,930) are drugs. It contains 3,775 sentences with two or more drugs, although only 2,044 sentences have at least one interaction. A total of 3,160 DDI were annotated at sentence level with the assistance of a pharmacist. The average number of interactions per document is 5.46 and per sentence 0.54.

### Detecting coordinate structures

Coordination is an extremely common grammatical phenomenon in biomedical texts. Since coordinate constituents are semantically close and usually they play the same syntactic and grammatical roles in a sentence, it is necessary to assemble them together [6]. For example, the following sentence contains three DDI:

• Aspirin may decrease the effects [of probenecid]*_PP_*, [sulfinpyrazone]*_NP_*, and [phenylbutazone]*_NP_*

In order to extract them, it is necessary to interpret the coordinate structure in it: *probenecid*, *sulfinpyrazone*, *and phenylbutazone*, in which the conjunction *and* coordinates the conjunct *probenecid* with *sulfinpyrazone* and with *phenylbutazone*.

Although a wide variety of structures can be conjoined, not all coordinations are acceptable. *Coordination of Likes Constraint* (*CLC*) [13] (also called *Law of Coordination ofLikes*) asserts that syntactically different categories cannot be conjoined. However, based on the corpus observation, this constraint is too restrictive for the kind of parsing provided by MMTx. For example, the above sentence demonstrates that being of the same syntactic category is too strong requirement for conjuncts in a coordinate construction, since a prepositional phrase, *of probenecid*, can be conjoined with two noun phrases: *sulfinpyrazone* and *phenylbutazone*. In fact, we have observed in the corpus that coordinate structures involving constituents with different syntactic categories are very common. Sometimes it is due to the fact that MMTx is not able to determine the syntactic type of a phrase, classifying it as an unknown phrase (that is, with the tag *UNK*).

Table 2 presents a set of syntactic patterns to detect coordinate structures, where the first row shows a pattern in which different syntactic types can be combined to detect coordination at the phrase level. An exception is made for verb phrases, since the coordination between a verbal phrase and another type of syntactic phrase is a coordination between clauses. Thus, the second pattern only allows to connect the verbal phrases with verbal phrases. Since this section focuses on coordination between phrases, we have only considered the coordinators *and*, *or*, *nor*, *and/or*, *as well as* as possible coordinators to link phrases. Table 2 also includes a syntactic pattern to detect correlative expressions such as *both midazolam and triazolam* (third row).

**Table 2:** Patterns to detect coordinate, correlative and appositive structures.

COORD	([*NP|PP|ADJ|UNK*],)* [*NP|PP|ADJ|UNK*] *CONJ* [*NP|PP|ADJ|UNK*]
	(*VP*,)* VP CONJ VP

CORRELATIVE	[*BOTH|EITHER|NEITHER*][*NP|PP|UNK*] [*AND|OR|NOR*] [*NP|PP|UNK*]
APPOSITIVE	[*NP*|*PP*|*UNK|APPOSITION*]
APPOSITION	*APPOSITIVE*(,)? (()? *MARKER* [*APPOSITIVE*(,)?]+ (*AND|OR*)? (*APPOSITIVE*)? ())?

### Identifying appositions

There are divergent views within Linguistics with regard to what is or is not an apposition (also called appositional or appositive structure). [14] and [15] restrict the category of apposition to coreferential noun phrases (called appositives) that are juxtaposed and refer to the same extralinguistic entity. [16] and [17] expand this definition with the inclusion of constructions such as clauses and sentences as possible elements of an apposition. [18] admits as apposition only those constructions which can be linked by a marker of apposition.

Although the above approaches provide insights into the category of apposition, they provide either an inadequate or an incomplete description of apposition. The objective of this work is not to provide formal and complete description of apposition, but rather to identify appositions, in particular, those that contain drugs. Thus, we only deal with appositions that are linked by a marker of apposition since this kind of apposition appears frequently in the sentences that contain DDIs. Markers are helpful clues for detecting these structures. The markers of apposition that we have used in this approach are: *such as*, *like*, *including*, *for example*, *e.g. and i.e.*. Appositions that are not linked by any marker are also frequent in scientific texts, however, the lack of markers makes the detection of this kind of apposition extremely difficult. Moreover, we have observed they hardly ever occur in expressions describing DDI.

We have defined a set of syntactic patterns in order to identify the appositions (see table 2). Appositions comprise at least two contiguous phrases, the second of which is marked by clues such as parentheses or markers. This second phrase may be a coordinate structure. The *APPOSITIVE* pattern allows to recognize the intervening elements in an apposition, that is, their appositives. This pattern matches a phrase type (provided by MMTx) or another apposition. In this way, the pattern is able to recognize nested appositions. Regarding the phrase types, it has not considered types such as *VP*, *CONJ*, *ADV*, or, *ADJ*, since our main focus is to recognize appositions containing drugs (drugs only appear in noun, preposition and unknown phrases). The *APPOSITION* pattern is used to recognize appositions. This pattern matches an intervening element *APPOSITIVE* followed by a marker and by one or more intervening elements expressed by coordinate phrases. Parentheses are also included in the pattern. Two different DDI can be extracted from the sentence:

• [Catecholamine-depleting drugs]*_NP_*, such as [Reserpine]*_NP_*, may have an additive effect when given [with beta-blocking agents]*_PP_*

(1) *Catecholamine-depleting drugs* with *beta-blocking agents*, and (2) *Reserpine* with *beta-blocking agents*.

Thus, it is essential to detect and resolve the appositions occurring in sentences, prior to the application of the lexical patterns responsible for DDI extraction. The appositions are firstly encapsulated and then unfolded when the relation is obtained by any lexical pattern.

### Clause splitting

Biomedical texts usually consist of extremely long sentences. Long sentences are usually complex or compound-complex sentences, that is, contain two or more clauses. For example, the following sentence contains two independent clauses (marked with *clause1* and *clause2*).

• Coadministration of CRIXIVAN and other drugs [that inhibit CYP3A4]*_rel_* [may decrease the clearance of indinavir]*_clause_*_1_ and [may result in increased plasma concentrations of indinavir]*_clause_*_2_.

Both clauses have the same subject: *Coadministration of CRIXIVAN and other drugs that inhibit CYP3A4*. This subject includes a relative clause (marked with *rel*) whose subject is *other drugs*.

Parsing-based and pattern-based approaches are inefficient to deal with complex and compound sentences. Parsers are usually trained in common English text corpora and are difficult to extend to new domains. For this reason, they usually fail particularly in biomedical complex sentences. Regarding the pattern-based methods, relations are possibly extracted incorrectly when patterns are matched beyond the scope of one clause or other kinds of grammatical units [6]. For example, the previous example contains a relative clause (*that inhibit CYP3A4*), which hinders the matching between the sentence and the *P*_8_ pattern (see Table 3). This section proposes an algorithm for clause splitting that aims to reduce the complexity of sentences in biomedical texts, in order to improve the performance of our pattern-based method for DDI extraction. Clause splitting is the task of dividing a complex or compound sentence into several clauses. The algorithm exploits syntactic and lexical information provided by MMTx. Once sentences have been split into clauses, a set of simplification rules is used in order to generate new independent sentences from the clauses. Finally, the lexical patterns defined by the pharmacist can be applied to the generated sentences in order to extract DDI.

**Table 3:** Lexical patterns to extract DDIs.

Id	Pattern
P1	DRUG *MODAL*? *ADV*? INTERACT*_syn_* WITH WORD_0..5_ (OF)? DRUG
P2	DRUG *MODAL*? *ADV*? INCREASE*_syn_* WORD_0..5_ (OF)? DRUG
P3	DRUG *MODAL*? *ADV*? DECREASE*_syn_* WORD_0..5_ (OF)? DRUG
P4	DRUG *MODAL*? *ADV*? ALTER_syn_ WORD_0..5_ (OF)? DRUG
P5	DRUG *MODAL*? BE *ADV*? INCREASE_syn_ WORD_0..5_ (BY)? DRUG
P6	DRUG *MODAL*? BE *ADV*? DECREASE_syn_ WORD_0..5_ (BY)? DRUG
P7	DRUG *MODAL*? BE *ADV*? ALTER_syn_ WORD_0..5_ (BY)? DRUG
P8	COADMINISTRATION OF DRUG (WITH|AND|PLUS) DRUG *MODAL*? *ADV*? [INCREASE*_syn_*|DECREASE*_syn_*|INTERACT*_syn_*|ALTER*_syn_*]
P9	COADMINISTRATION OF DRUG (WITH|AND|PLUS) DRUG *MODAL*? *BE*? *ADV*? RESULT*_syn_* (TO|WITH|IN) [INCREASE*_syn_*|DECREASE*_syn_*|INTERACT*_syn_*|ALTER*_syn_*]
P10	CAUTION *MODAL*? *ADV*? *BE*? USED WHEN DRUG *WORD*? (WITH|AND|PLUS) DRUG *BE*? ADMINISTERED*_syn_ CONCURRENTLY*?
P11	PATIENTS TREATED (WITH)? DRUG (WITH|AND|PLUS) DRUG (CONCURRENTLY)? MODAL BE OBSERVED*_syn_*
P12	INTERACTION (OF|BETWEEN) DRUG (AND|WITH|PLUS) DRUG MODAL? (BE)? WORD_0..3_ (OBSERVED*_syn_*|INCREASE*_syn_*|DECREASE*_syn_*|ALTER*_syn_*)

We now explain how the sentences are broken into clauses. First of all, it is necessary to ensure that the sentence is actually a compound or a complex sentence. It is not enough to check that there is some coordinator or subordinator in the sentence since sometimes they do not function like connectors between clauses, but as prepositions, adverbs, etc. A possible heuristic is to count the number of verb phrases included in the sentence. To give a definition of verb phrase is not an easy task. In fact, linguists have not even reached an agreement on what the verb phrase should include: only the words that are verbs, or also the complements of the verb. While the generative grammarians propose that a verb phrase consists of various combinations of the main verb and any auxiliary verbs, plus optional specifiers, complements, and adjuncts (for example, *Anagrelide* [*may interacts with any of these compounds*]*_VP_*), for functionalist linguists the verb phrases consist only of main verbs, auxiliary verbs, and other infinitive or participle constructions [19] (for example, *Anagrelide* [*may interacts*]*_VP_* [*with any of these compounds*]*_PP_*). We have decided to adopt the last definition, that is, we define a verb phrase as a syntactic structure that is composed of a main verb and, optionally, of auxiliary and modal verbs, but the complements are excluded of this structure. Unfortunately, MMTx offers an even simpler definition of verb phrase, because MMTx labels each verb as a *VP*. Forms of *to be* are labeled as *V/_be_*. In order to group the main verb, its auxiliary or modal verbs, as well as its adverbial complements in the same verb phrase, we define the VP-pattern as: [VP|V/*_be_*|VPG] _(V/_*_be_*_)? (NOT)? (ADV)?_ (VP|V/*_be_*|VPG)? (TO VP)?. The *VP-pattern* is applied to sentences in order to merge their adjacent verb phrases into an extended verb phrase. If a sentence contains two or more extended VPs, then we can conclude that it is a complex or compound sentence. However, if a sentence only contains an extended *VP*, it is a simple sentence despite containing any conjunction. First column in Table 4 shows some sentences parsed by MMTx, while the second column shows the result of applying our *Vp-pattern* to them.

**Table 4:** How does MMTx label the verb phrases?

Verb phrases detected by MMTx	Verb phrases joined by the VP-pattern
[Formal drug interaction studies]*_NP_* [have]*_VP_* [not]*_ADV_* [been]*_V/be_* [conducted]*_VP_* [with ORENCIA.]*_PP_*	[Formal drug interaction studies]*_NP_* [**have not been conducted**]*_VP_* [with ORENCIA.]*_PP_*
[The combination]*_NP_* [of methotrexate]*_PP_* [with acitretin]*_PP_* [is]*_V/be_* [also]*_ADV_* [contraindicated]*_VP_*	[The combination]*_NP_* [of methotrexate]*_PP_* [with acitretin]*_PP_* [**is also contraindicated**]*_VP_*

Once it has been determined that the sentence contains two or more clauses, the following step is to determine the type of sentence. Such information will be very useful in detecting the clause boundaries. In the English language, a *compound sentence* is composed of two or more independent clauses joined by a conjunction that can be a coordinator (coordinating conjunction: *for*, *and*, *nor*, *but*, *or*,*yet*, *so*), a correlative conjunction (*both*, *either*, *whether... or; not only... but also*) or an independent marker word (*however*, *moreover*, *furthermore*, *consequently*, *nevertheless*, *therefore*). Semicolons and commas can also function as conjunctions. If an independent marker occurs at the beginning of the sentence, then a semicolon or a comma should separate the clauses. If the second independent clause starts with an independent marker, then a semicolon or a comma is needed before the marker [20]. The independent markers can also occur in simple sentences, as in the following sentence: *However*, *initial dose modification is generally not necessary*.

A *complex sentence* has an independent clause joined with one or more subordinate clauses. Subordinate clauses contain both a subject and a verb, but do not express a complete thought. A complex sentence always has a relative pronoun (*who*, *that*, *which*, *whoever*, *whom*, *whomever*, *whose*, *whichever*, *whatever*) or a subordinator (*after*, *although*, *as*, *as if*, *because*, *before*, *even if*, *even though*, *if*, *in order to*, *since*, *though*, *unless*, *until*, *whatever*, *whether*, *when*, *whenever*, *while*.) that links the clauses. If the complex sentence begins with a subordinator, that is, the subordinate clause is at the beginning of the sentence, then the subordinate clause should end with a comma. On the other hand, if the independent clause is attached at the beginning of the main sentence and the subordinator is in the middle, then no comma is required [20].

Taking into account the above clues, we initially defined a set of lexical patterns for detecting clauses boundaries in compound and complex sentences (see Table 5). Relative clauses are a especial case, since, they often appear in the middle of a main clause, splitting it into two parts. If a sentence matches some of these patterns, then its clauses can be easily extracted from the matching.

**Table 5:** Initial patterns for clause splitting.

Compound sentences	CLAUSE_1_(,|;)? [*indepMarker|coordinator*|;|,] CLAUSE_2_
	*indepMarker*(,)*?* CLAUSE_1_[,|;] CLAUSE_2_

Complex sentences	*depMarker*(,)*?* CLAUSE*_subordinate_*, CLAUSE*_main_*

		CLAUSE*_main_* [*depMarker*|*;*|,] CLAUSE*_subordinate_*

Relative Clauses	*relativePronoun* (NP|PP|UNK|ADJ|APOS|COORD)? VP [NP|PP|UNK|ADJ|APOS|COORD]

However, these patterns are not always enough. Determining where a clause ends is not always a trivial task, since there might be commas or conjunctions internal to the clause. Moreover, some conjunctions can also function as prepositions (for example *for*) or as adverbs (for example *yet*, *so*). The problem regarding adverbs is easily resolved (at least in most of cases) because MMTx labels them as *CONJ* phrases when they function as coordinators (though sometimes MMTx mistakes the phrases or is not able to determine the types). The previous identification of appositions and coordinate structures allows to reduce the number of commas and conjunctions internal to a clause. However, for each comma or coordinator not included in any apposition or coordinate structure, it is required to know whether the clause ends or not in it. Therefore, the above patterns have been replaced with a set of heuristics based on the observation of fifty compound and complex sentences. These heuristics are encoded in algorithm 1.

In a few words, the algorithm works as follows. the input of the algorithm is the sentence in which its verb phrases have been joined by the VP-pattern. First of all, the algorithm must check that the sentence contains two or more clauses. Then, the sentence is reviewed while it contains any separator marker. A separator marker can be a coordinator, a independent marker, a dependent marker, a semicolon or a comma. The coordinators and subordinators must be labeled by MMTx as *CONJ* phrases, otherwise, they are not considered as conjunctions. Then, the algorithm iteratively finds candidate clauses, that is, a substring of the sentence between markers. If the candidate clause contains a verb phrase, then it is considered as clause. The algorithm is able to decide the kind of clause, that is, independent or subordinate.

#### Rules for sentence simplification

Once appositions and coordinate propositions have been recognized, and compound and complex sentences have been split into clauses, it is possible to apply a set of rules for sentence simplification. These rules allow to simplify the complex and compound sentences in simple sentences. Then, the pattern-based approach for DDI extraction will be applied to these simpler sentences.

We have adapted some of the simplification rules presented in [4]. This work also recognized relative clauses, apposition, coordination and subordination, however its goal was not relation extraction, but to provide syntactic simplification of sentences for improving the performance of NLP applications such as text summarization or machine translation. [4] proposes seven simplification rules to generate new simplified sentences from the clauses of the complex and compound sentences. Table 6 presents the rules adapted in our approach and some sentences broken up into simpler sentences by these rules. The following list shows examples of how the simplification rules split complex and compound sentences:

**Table 6:** Rules to generate new simplified sentences from the clauses. The clause CLAUSE*_REL_*_(_*_NP_*_)_ means that it is attached to the noun phrase NP.

Simplification Rules	Generated sentences
MARKER(,)? CLAUSE_1_, CLAUSE_2_	(1) CLAUSE_1_ (2) CLAUSE_2_
CLAUSE_1_(,)? MARKER CLAUSE_2_	(1) CLAUSE_1_ (2) CLAUSE_2_
CLAUSE_1_ NP CLAUSE*_REL_*_(_*_NP_*_)_ CLAUSE_2_	(1) CLAUSE_1_ NP CLAUSE_2_ (2) NP CLAUSE*_REL_*_(_*_NP_*_)_

• [Because]*_MARKER_* [busulfan is eliminated from the body via conjugation with glutathione]_*CLAUSE*_1__ [use of acetaminophen prior to (72 hours) or concurrent with BUSULFEX may result in reduced busulfan clearance based upon the known property of acetaminophen to decrease glutathione levels in the blood and tissues]_*CLAUSE*_2__.

• [Although]*_MARKER_* [the interactions observed in these studies do not appear to be of major clinical importance]_*CLAUSE*_1__, [BREVIBLOC should be titrated with caution in patients being treated concurrently with digoxin, morphine, succinylcholine or warfarin.]_*CLAUSE*_2__

• [Trimeprazine also decreases the effect of heparin and oral anticoagulants,]_*CLAUSE*_1__ [while]*_MARKER_* [MAOIs can increase the effect of trimeprazine.]_*CLAUSE*_2__

The following sentence (containing a relative clause) is transformed into the two simpler sentences (1) and (2):

• Since the excretion of oxipurinol is similar to that of urate, uricosuric agents, *which increase the excretion of urate*, are also likely to increase the excretion of oxipurinol and thus lower the degree of inhibition of xanthine oxidase.

1. Since the excretion of oxipurinol is similar to that of urate, uricosuric agents are also likely to increase the excretion of oxipurinol and thus lower the degree of inhibition of xanthine oxidase.

2. Uricosuric agents (which) increase the excretion of urate.

### Lexical patterns for DDI extraction

Despite the richness of natural language expressions, in practice, DDI are often expressed by a limited number of constructions. This fact favors the use of patterns as an excellent method for their extraction. Based on her professional experience and the corpus observation, our pharmacist defined a set of lexical patterns (see Table 3) to capture the various language constructions used to express DDI in pharmacological texts. Moreover, the pharmacist provided a set of synonyms for the verbs that can indicate a possible DDI (see Table 7).

**Table 7:** Auxiliary patterns.

**MODAL**=[CAN|COULD|MAY|MIGHT|SHOULD|MUST|HAVE|HAS|HAD]
**BE**=[IS|ARE|WAS|WERE|BE|BEEN]
**ADV** is any adverbial except ’NOT’. For example, also,potentially, etc.
**INTERACT***_syn_*=[INTERACT|INTERFERE]
**INCREASE***_syn_*=[AUGMENT|ELEVATE|ELEVATE|ENHANCE|EXACERBATE|EXTEND|GO_UP|INCREASE|INTENSIFY|POTENTIATE|PROMOTE|PROLONG|RAISE|RISE|STIMULATE]
**DECREASE***_syn_*=[DECREASE|DIMINISH|LESSEN]
**ALTER***_syn_*=[ACCELERATE|ANTAGONIZE|ALTER|CHANGE|INDUCE|INFLUENCE|INHIBIT]
**RESULT***_syn_*=[RESULTS|ASSOCIATED|SHOWN|RESULTED|OBSERVED|DETERMINED]
**WHEN**=[WHEN|IF|WHETHER]
**ADMINISTERED**=[CO-ADMINISTERED|COADMINISTERED|ADMINISTERED|TAKEN|GIVEN|USED|EMPLOYED]
**PATIENTS**=[PATIENTS|SUBJECTS|
**TREATED**=[TAKEN|TREATED|RECEIVING|TAKING]

## Results

This section explains in detail the experiments that we have carried out to evaluate the performance of the DDI extraction. We consider as baseline system, so called *allDDIs*, the case in which every pair of drugs that co-occur in a sentence are assumed to interact. This baseline yields the maximum recall, but low precision (11%) and a baseline F-measure of 19%. The most basic experiment in which neither coordinations, appositions nor clauses are tackled, that is, the lexical patterns are directly applied to the text of sentences. First of all, sentences are parsed by MMTx and drug names are identified by the DrugNer system [21]. Then, only those sentences that contain two or more drug names are selected and the drug names are replaced by the label *DRUG._index_*, where *index* shows the order of each drug in the list of drugs that occur in sentence. Finally, the set of lexical patterns is applied to the text of the sentence.

When a sentence has been correctly matched with a pattern, it must be checked if the matching string includes the negative adverb (*NOT*). If it is not included, then a possible interaction has been found. Drug names that occur in the matching are retrieved, and the pair of drug names is proposed as a DDI.

In the second experiment, appositions and coordinate structures are identified in text by the set of syntactic patterns above described. The lexical patterns were modified to consider these structures, that is, they are extended for including the labels *APPOSITION* and *COORD* as possible elements participating in the interactions. Thus, for this experiment, *DRUG*:= [*DRUG|APPOSITION|COORD*]. The procedure of matching pattern for this experiment is explained in algorithm 2.

Table 8 shows the global and individual pattern performance. The basic experiment achieves a reasonable precision (67.30%), but very low recall (14.07%). The average number of DDI detected by each pattern is 35.5 (the total number of DDI in the DrugDDI corpus is 3,160). Regarding the individual pattern performance, the highest recall is achieved by the pattern *P2* and the highest precision by the pattern *P8*. Regarding the second experiment, recall is improved by the inclusion of the appositions and coordinate structures, however, precision is lower. The average number of DDI detected by each pattern is 64.83. The pattern *P2* still achieves the highest recall, and the highest precision is obtained by the pattern *P10*.

Therefore, the detection of these structures achieves to improve the recall (almost 12%) with a significant decrease in precision of almost 19%. This decrease can be attributed to the errors introduced during syntactic processing.

**Table 8:** Results.

	Patterns			Coord+Apos			Coord+Apos+Clauses		
**Id**	**P**(%)	**R**(%)	**F***_β_*_=__1(%)_	**P**(%)	**R**(%)	**F***_β_*_=_* *_1 (%)_	**P**(%)	**R**(%)	**F***_β_*_=_* *_1 (%)_

P1	60.71	0.56	1.11	59.17	2.35	4.51	59.17	2.35	4.51
P2	69.51	3.77	7.15	54.78	7.00	12.42	55.75	6.41	11.50
P3	53.28	2.15	4.13	44.74	3.93	7.23	46.18	4.00	7.36
P4	68.64	2.68	5.15	52.67	4.56	8.39	52.69	4.53	8.34
P5	79.17	0.63	1.25	48.19	1.32	2.57	52.00	1.29	2.51
P6	60.00	0.30	0.59	39.13	39.39	0.43	0.85	0.30	0.59
P7	77.42	0.79	1.57	60.00	0.99	1.95	58.33	0.93	1.82
P8	100.00	0.50	0.99	57.45	0.89	1.76	52.54	1.02	2.01
P9	73.81	1.02	2.02	68.18	1.98	3.85	68.18	1.98	3.85
P10	85.71	0.20	0.40	50.00	73.33	0.36	0.72	0.10	0.20
P11	87.88	0.96	1.90	19.69	1.26	2.36	20.21	1.29	2.42
P12	47.06	0.53	1.05	35.19	0.63	1.23	35.19	0.63	1.23
GLOBALS	67.30	14.07	23.28	48.69	25.70	33.64	48.89	24.81	32.92

The last experiment combines the detection of appositions, coordinate structures, clause splitting and simplification rules. First of all, appositions and coordinate clauses are detected by applying the previous described procedure (algorithm 2) step by step until the sixth step. Then, the algorithm 1 is applied to sentences in order to split the complex and compound sentences into their clauses. New sentences are generated from these clauses by the simplification rules. Finally, the previous procedure of matching pattern (algorithm 2) is applied to these new sentences from the seventh step.

As a preliminary step we performed an evaluation of linguistic structures resolution on a set of fifty sentences, which were randomly selected and manually tested with the assistance of a linguist. Results are shown in Table 9. We observed that most of the errors were due to tagging and parsing mistakes made by MMTx. Both the error analysis and the improvement of MMTx are two issues that are out of scope of this work. Clause splitting is a very complex task, which consists of three tasks: identifying clause starts, identifying clause ends, and finding complete clauses (many of them may be nested clauses). The nesting of clauses is very common in biomedical texts. Our method mainly fails to deal with the resolution of nested clauses. However, though it obtains lower results, we believe that it is a good initial approximation for clause splitting in the biomedical domain.

**Table 9:** Evaluation of linguistic structures resolution.

Structure	TP	FN	FP	P	R	F
Coordinate	24	14	0	1	0,63	0,77
Appositions	11	2	0	1	0,85	0,92
Relatives	6	3	0	1	0,67	0,8
Rest of Clauses	16	7	0	1	0,7	0,82

Results on DDI extraction are shown in Table 8. While the inclusion of appositions and coordinate structures achieved to improve the recall, and therefore, the f-measure, the detection of clauses did not improve overall performance.

Although we are aware that the syntactic simplification evaluation is quite shallow to reach definite conclusions about performance it seems to point out that the chaining of errors may have a larger impact. In addition, many interactions occurring in complex sentences often span several clauses (for example, *The Cmax of norethindrone was 13% higher when it was coadministered with gabapentin*). The lexical patterns are not able to capture these interactions that would require a more complex semantic interpretation.

## Conclusions

In this paper, we have proposed a hybrid method that combines the resolution of complex linguistic constructions and pattern matching.

Regarding the resolution of the linguistic constructions, as it was pointed out in the Results section, most of the errors are due to mistakes introduced in the MMTx level and the difficulty of resolving nested clauses, so frequent in biomedical texts. Also, we are aware that our clause splitting method is too simplistic to deal with the complexity of biomedical sentences.

Another shortcoming of our current approach is that negation has been only slightly addressed. Although the following sentence matches the pattern *P1*, it does not represent any interaction:

• While studies have not shown*DRUG*_1_*interact with DRUG*_2_, caution should be exercised.

A deeper treatment of negation should discover that the phrase *studies have not shown* have a larger scope that includes the interaction.

Future directions include trying to identify and resolve the errors of MMTx and analyzing the effect on the DDI extraction performance, improving our clause splitting algorithm, proposing new suitable simplification rules to regenerate the simple sentences from clauses, checking what occurs if the resolutions are applied in a different order, studying the utility of other corpora such as Genia-GR [22] or Penn Treebank [23] and other parsers such as Stanford [24] or MiniPar [23], and increasing the size of the corpus and annotating it with these linguistic constructions. In addition, we will carry out a more exhaustive treatment of negation and modality in sentences. We will also study the overall contribution of our anaphora resolution approach [25] to the broader task of DDI extraction.

Concerning the performance in the extraction of DDI, the variability of natural language expression makes it difficult for our method to accurately detect all semantic relations occurring in text since sentences conveying the same relation may be composed lexically and syntactically differently. Inversely, sentences that are lexically common may not necessarily convey the same relation. Thus, our lexical patterns are not enough to identify many of the interactions. Future work will include the application of bootstrapping techniques to find additional patterns like the SPINDEL system [26]. Continuing the work presented in [27], we also plan to apply advanced machine learning techniques to extract DDIs.

## Competing interests

The authors declare that they have no competing interests.

## Authors' contributions

IS carried out the study of the related work, developed the design and implementation of the system and participated in its evaluation. PM carried out the study of the related work, participated in the design and coordination of work, and helped draft the manuscript. CP designed the arquitecture of the system, and took part in the implementation and evaluation of the system. All authors read and approved the final manuscript.
